# Comparable responses to a wide range of olfactory stimulation in women and men

**DOI:** 10.1038/s41598-023-35936-5

**Published:** 2023-06-03

**Authors:** Moa Lillqvist, Anna-Sara Claeson, Marta Zakrzewska, Linus Andersson

**Affiliations:** 1 Department of Psychology, Umeå University, Umeå, Sweden; 2 Department of Clinical Neuroscience, Karolinska Institute, Solna, Sweden

**Keywords:** Psychology, Human behaviour

## Abstract

The evidence for differences between women and men in terms of olfactory abilities is contradictory. We analyzed women and men’s performance and reactions to a wider range of odour exposure outcomes than usually studied, to assess possible differences and similarities between sexes. Measures of sensitivity and sensory decision rule were established in 37 women and 39 men. Perceptual, cognitive, symptom-related and autonomic nervous system (skin conductance level and heart-rate variability) reactions were also assessed during extended ambient odour exposure, as well as participants’ self-rated chemical intolerance. Bayesian analyses consistently revealed greater support for sex-related similarities than differences, suggesting that women and men perform and react comparably not only in terms of basic olfactory measures, but also to environmental odour exposure mimicking everyday situations.

## Introduction

Women have generally been described as having a more acute sense of smell than men^1–4^. This idea has been perpetuated throughout history, and can be readily traced through literature and other cultural expressions^5,6^. Vision and hearing, the distal senses, have been associated with masculinity and rationality, necessary for political and logistical endeavours. The more proximal senses of smell, taste and touch, have in comparison, been regarded as more corporeal in nature, and affiliated with the traditionally feminine domain of home and hearth^5^. The nose itself is a ‘feminine organ; delicate and leaky’ according to seventeenth century medical literature^7^.

Numerous contemporary studies support the assumption of female olfactory superiority. Women have been reported to outperform men in absolute detection^3,4,8–10^, as well as in discrimination and identification tasks^3,11^. In a meta-analysis by Sorokowski et al.^3^, a female olfactory advantage was supported in all three aforenoted domains. Women also report being adversely affected by odours to a greater degree than men^12,13^.

A prominent explanation is that there are sex-specific evolutionary advantages to having a fine sense of smell. According to the embryo protective hypothesis, a woman’s ability to identify and avoid toxins through the chemical senses constitutes an important line of defense that protects the embryo during vulnerable stages of pregnancy, resulting in healthier offspring^14,15^. Olfactory acuity has also been argued to co-vary with levels of circulating gonadal hormones, which would explain fluctuations in odour perception throughout the menstrual cycle and the stages of pregnancy ^16^. Similarly, the evolutionary/hormonal framework has also been used to explain why women tend to report more distress or higher negative valence by unpleasant odours than men^1,3,5,16–18^. On the other hand, the notion of female superiority in olfaction has also been challenged by studies where no significant differences in the basic olfactory function between men and women have been found^11,19–24^. Guarneros et al.^21^ found no differences between men and women in all three dimensions of detection, discrimination and identification. Öberg et al.^24^ found no difference in detection or discrimination. Larsson et al^11^ found no difference in detection and identification., Cain and Gent^19^, Lundström et al^23^ and Kern et al^22^ in detection and lastly, Good et al.^20^ found no difference between men and women when it comes to identification tasks.

Alternative explanatory frameworks suggest that differences between women and men may be due to varying life experiences, gender roles and gendered expectations on olfactory performance. Ferdenzi et al.^25^ studied children’s behavior related to olfaction and found gendered differences in attention to odours from an early age. Girls were more attentive and reactive to odourous cues than boys, which Ferdenzi et al.^25^ suggested to be an effect of social expectations. Brand and Millot^1^ argued along similar lines that women encounter odours more often than men do, and thus have greater experience of processing olfactory cues. Andersson et al^26^ reported physiological results showing that women and men differed in olfactory event-related potentials (i.e., a measure of central nervous system processing of odours), but only in a task where olfactory cues were to be attended but not when the participants were asked to ignore the stimuli. This suggests a difference between women and men at later, rather than earlier and arguably less malleable sensory processing stages.

It is thus possible that women are on average more culturally exposed to using the sense of smell than men^1,25^ or that expectations guide attention to chemosensory stimuli differently in women and men. Women tend to both rate themselves as being more attentive to odours in their everyday lives and that smell is more important to them than men do^23,25,27^ and they also rate their olfactory abilities higher^4^. Nováková et al^17^ also found that the conformity to gender roles in children could predict olfactory abilities, especially in boys.

From a more overarching perspective, Danielsson et al.^28^ argue that gender and cultural context continuously shape perception and behaviour. Acting or reacting in ways that are considered ‘feminine’ or ‘masculine’ in particular contexts can be considered an adaption to prevailing gender norms, which influences perception. Such arguments are similar to ‘stereotype threat’ research frameworks, where differences between groups assumed to be innate has been shown to stem from cultural / societal expectancies or roles^29^.

The nature or existence of sex or gender related differences in olfactory ability is thus still an open field of inquiry, with differing and sometimes opposing empirical results and theoretical explanations.

Here we expand the study of possible similarities and differences by assessing performance and reactions to a wide repertoire of olfactory and related tests in women and men. We establish measures of both sensitivity (i.e. olfactory acuity) and criterion (i.e. propensity to judge an odour as present or absent when uncertain) using a method of constant stimuli. This procedure has been shown to establish threshold data very close to professional grade olfactometry, and not only a score value where the concentration of the odourant reaching the nose is less clear^30^. Further, we investigate reactions to ambient odour exposure inside a chamber that allows whole body exposure. The rationale for using a chamber is to mimic everyday chemical exposure, where the odour stimulus is present in the ambient air. This procedure also implies a naturalistic way of inhaling the odourant, rather than through e.g., active sniffing (like in the case of bottles) or through a forced airflow through the nose (like the case of dynamic olfactometry).

While seated in the chamber, participants repeatedly rate the perceptual properties of the odour exposure in terms of intensity and valence^31^, but also to what degree the exposure influences their ability to concentrate. The latter measure is important as the distractability of odours is often reported as a core problem among individuals who react negatively to chemical stimuli^32^. In a similar vein, we asked for possible symptoms following the extended exposure.

In addition to the ratings, the study included cognitive tasks related to inhibition and working memory. The former task was included to probe whether odour stimulation places a different load on inhibition among women and men, in a way that is similar to previous reports of inhibitory differences between individuals who sensitize or habituate to odours^33^. The working memory task was included to probe performance while exposed in more general terms. Pacharra et al^34^ reported that they found no effects on cognitive functioning during an odour exposure with during a similar working memory task.

Given that odour exposure may be a salient stressor, we also recorded tonic electrodermal activity (skin conductance level; SCL) and heart-rate variability (HRV) as measures of autonomic nervous system activity during exposure. Possible differences between women and men in these outcomes could thus possibly hint at different regulatory/homeostatic reactions to exposure. Finally, we ask for self-rated intolerance to odours in everyday life. The measures in this study were thus chosen to reflect important odour-related outcomes to complement those commonly assessed in studies of sex-related differences in olfaction.

Using Bayesian methods, we investigated possible differences in reactions to olfactory / chemosensory stimulation between women and men using the following outcome measures: (1) olfactory sensitivity, and (2) criterion; (3) perceptual and (4) symptom ratings during exposure; (5) performance on cognitive tasks during exposure; (6) autonomic reactions during exposure in the form of SCL and HRV; and finally (7) self-reported problems with odours in everyday life.

## Methods

### Participants

Adult participants (37 women, mean age = 38, SD ± 14; 39 men, mean age = 34, SD ± 12) were recruited through public advertisement. The advertisement was conducted throughout public spaces in the northern Swedish city of Umeå to enroll a sample with demographic characteristics more comparable to the population than e.g., a student sample. Smoking and pregnancy were exclusion criteria. The study was conducted in accordance with the Helsinki Declaration and approved by the Umeå Regional Ethics Board (ref.: 2015/99-31Ö and 2016–31-32Ö).

### Stimuli, instruments, and tests

#### Assessment of olfactory acuity and sensory decision rule

Non-parametric measures of olfactory sensitivity (A) and criterion (ln(b)) were established using a method of constant stimulus procedure^35^, and calculated according to Zhang and Mueller^36^. Stimuli consisted of 60 ml dilutions of the odourant *n*-butanol (99.7% VWR) mixed with water in 500 ml glass flasks, equivalent to dilution steps 6 through 12 described by Cain^37^. Blank stimuli consisted of 60 ml pure tap water. Each dilution step was presented 12 times, whereas blanks were presented 48 times, for a total of 132 presentations in random order. The inter-stimulus intervals were at least 20 s, with a two-minute break after each 14:th flask. The *n*-butanol flasks were used for no more than three days before exchanging them for new batches (refrigerated when not in use). Blank flasks were changed every day.

#### Extended olfactory exposure

An exposure chamber (Fig. 2) was utilized to assess reactions to extended olfactory exposure. Carbon filtered air was fed into the chamber through an inlet close to the floor and ventilated out through a ceiling vent. Nebulized *n*-butanol was added to the filtered air at a concentration controlled by a syringe pump. The concentration inside the chamber was repeatedly controlled through gas chromatographic analyses and did not fluctuate more than 10%. The exposure session began with 11 min. Of clean air exposure (blank/sham), followed by 9 min of increasing concentration and 26 min at a 11.5 mg/m^3^ plateau.

#### Perceptual and symptom ratings

Participants rated the intensity, valence (pleasantness / unpleasantness) and impact on concentration (positive / negative influence on the ability to concentrate) of the *n*-butanol exposure using a Borg CR-100 scale. This is a category rating scale containing both numbers and verbal anchors: Nothing, 0; minimum, 1.5; extremely weak, 2.5; very weak, 6; weak, 12; moderate, 25; strong, 45; very strong, 70; extremely strong, 90; near maximal, 100. Numbers above 100 are not labelled but can be used. Unpleasantness and negative influence on ability to concentrate was entered as negative values for valence and concentration, respectively.

The ten most frequently reported symptoms by persons with intolerance to chemicals/odours ^38^ were also rated using the Borg CR-100 scale; eye irritation, nasal mucosal irritation, skin irritation, throat irritation, shortness of breath, concentration difficulties, dizziness, tiredness, headache and nausea. The mean of these ten symptoms were used as a composite score in the statistical analysis. All ratings were made using pen and paper, following a prompt on a computer screen.

#### Cognitive tasks

A Stroop task was used as a general measure of inhibition/interference^39^. Participants read out loud the font color of incongruous words as rapidly as possible (e.g., the word “blue” in red font was to be verbalized as “red”). An image with 84 Stroop words were presented for 45 s. Answers were recorded through a microphone. Performance was calculated as number of correctly labeled colors minus non-corrected errors.

A 3-back task was used as a general measure of working memory capacity / updating^40^. A sequence of 30 numbers (1–9) were presented on a computer screen, each with a duration of 1.3 s, and an inter-stimulus interval of 0.2 s, for a total duration of 45 s. The task was to indicate whether or not the current number was the same as that presented three numbers back (i.e. one button for yes, another for no). Performance was calculated as proportion of correct yes responses (out of 12).

#### Autonomic nervous system recordings

A BIOPAC MP100 system was used to record electrodermal activity (EDA) and electrocardiograms (ECG). EDA was recorded at 1000 Hz using a wired transducer (TSD203) coated with isotonic electrode gel attached to the distal phalanges of the non-dominant middle and index fingers. The signal was filtered offline with a 1 Hz low pass filter, after which a duplicate 0.05 Hz high pass filtered waveform was subtracted to remove phasic skin conductance responses. Mean skin conductance level (SCL) was extracted as a measure of tonic EDA from the remaining waveform, providing nine 5-min segments.

ECG was recorded at 1000 Hz using disposable electrodes (EL503) attached to the non-dominant wrist and corresponding ankle. Heart-rate variability in the form of root mean square of the normal-to-normal interbeat intervals (RMSSD) was extracted into nine 5 min segments using Kubios 2.1. Artifact correction was done manually. Uncorrected or missing R peaks (mean number = 3.1, SD = 8.9 per recording) were corrected using the software’s algorithm.

#### Self-rated chemical intolerance

The Chemical Sensitivity Scale^41^ was used to assess self-rated affective and behavioral consequences of everyday chemical exposure. It consists of statements such as “At movies, other persons’ perfume and aftershave disturb me” that are rated regarding degree of agreement with the statement, importance, or frequency.

#### Procedure

After having expressed their interest, participants were given information and a consent form through mail, and an invitation to schedule themselves for testing. The study setup was also explained on site and initiated after a consent signature. During the first day, participants’ olfactory acuity and sensory decision rules were established. Further, they also performed a first set of Stroop and 3-back tasks.

**Figure 1:**
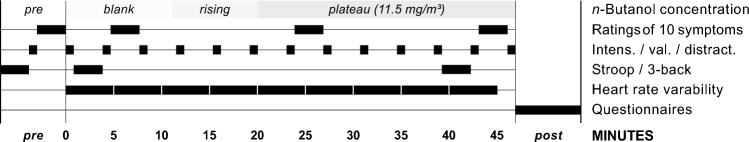
Overview of the exposure sequence.

At the second day of testing, participants once more performed the Stroop and 3-back tasks, after which they were fitted with electrodes and seated in the exposure chamber. They repeatedly rated the properties of the odour—before closing the chamber door, three times during blank, three times during rising, and seven times during plateau exposure. Further, they rated symptoms before, during blank, and two times during plateau exposure. Stroop and 3-back were performed both in the beginning and the end of the exposure, and autonomic measures were collected repeatedly. After the exposure session, participants filled out questionnaires (cf. Fig. 1).

#### Statistical analyses

We analyzed data from women and men using used JASP (version 0.17.1, jasp-stats.org). As we hypothesised that there would be no differences between men and women, we performed Bayesian analysis, which allows evaluating evidence in favour of the null hypotheses. We report the Bayes factor (BF) in favor of the null hypothesis (BF_01_, no differences between men and women) using Jeffreys terminology^42^), and estimates of the difference between sexes along with 95% credibility intervals (Cis). For one-time evaluations (olfactory acuity and self-rated chemical intolerance) we used independent samples Bayesian t-tests (with a default Cauchy prior with scale = 0.707). Evaluations repeated multiple times (perceptual and symptom ratings, cognitive tasks and autonomic nervous system recordings) were analyzed using the Bayesian repeated measures analysis of variance (ANOVA) with default priors: r scale set to 1 for random effects, and to 0.5 for fixed effects.

The Bayesian repeated measures ANOVAs included a null model with the effect of subject and random slopes for all repeated (within subject) measures factors. We also added the effect of the other factors: time of measurement (Time, 14 ratings), Sex (Men and Women) and an interaction between these two factors. The inferences about the effects of Sex, and a Time * Sex interaction were based on all models which included the given effect. For each analysis, we report a BF (BF_excl_) in favor of *not* including the effect of Sex (or a Time * Sex interaction) when predicting given ratings. Thus, the reported BF reflects evidence for the null hypothesis (BF_01_, no differences between men and women). To give an idea of the size of the difference between men and women, we report estimates from repeated measures ANOVA including all effects (Time, Sex and the interaction between the two) along with 95% credible intervals (CI), either in the results section or in Supplementary materials (Table S1-S8).

## Results

Table 1 shows descriptive statistics for all variables of interests separately for men and women.

**Table 1:** Descriptive statistics of the variables in the data.

		N	Missing	Mean	SD	Min	Max
Age	M	**3**9	0	34.46	11.51	18.0	66.00
	W	37	0	37.57	14.36	21.00	63.00
Olfactory sensitivity (A)	M	39	0	0.81	0.09	0.56	0.96
	W	37	0	0.79	0.08	0.62	0.92
Criterion (ln(b))	M	39	0	0.22	0.57	− 0.99	1.57
	W	37	0	0.25	0.52	− 0.50	1.29
Rated intensity	M	38	1	14.13	8.99	1.07	38.21
	W	35	2	17.14	11.36	2.64	49.93
Rated valence	M	38	1	− 5.41	9.68	− 36.29	16.79
	W	34	3	− 5.69	15.03	− 45.07	28.07
Rated impact on ability to concentrate	M	38	1	− 3.96	7.15	− 36.29	4.64
	W	34	3	− 5.72	13.38	− 44.07	27.07
Rated cardinal symptom	M	38	1	6.23	4.73	0.30	15.60
	W	35	2	6.92	6.27	0.00	22.43
Stroop task score	M	31	8	51.94	10.33	26.50	71.00
	W	29	8	50.78	9.20	30.75	72.50
3- back score	M	36	3	5.34	1.77	1.25	8.75
	W	34	3	5.40	2.29	− 0.75	10.50
Skin conductance level	M	38	1	0.39	0.16	0.15	0.79
	W	35	2	0.34	0.17	0.09	0.86
Hear rate variability	M	36	3	76.57	12.29	53.66	106.07
	W	34	3	77.24	11.47	55.24	103.26
Self-rated chemical intolerance	M	39	0	50.69	17.34	16.00	85.00
	W	37	0	55.57	15.11	23.00	82.00

*M* Men, *W* Women, *SD* Standard deviation, *Min* Minimum, Max Maximum.

### Olfactory acuity and sensory decision rule

**Figure 2:**
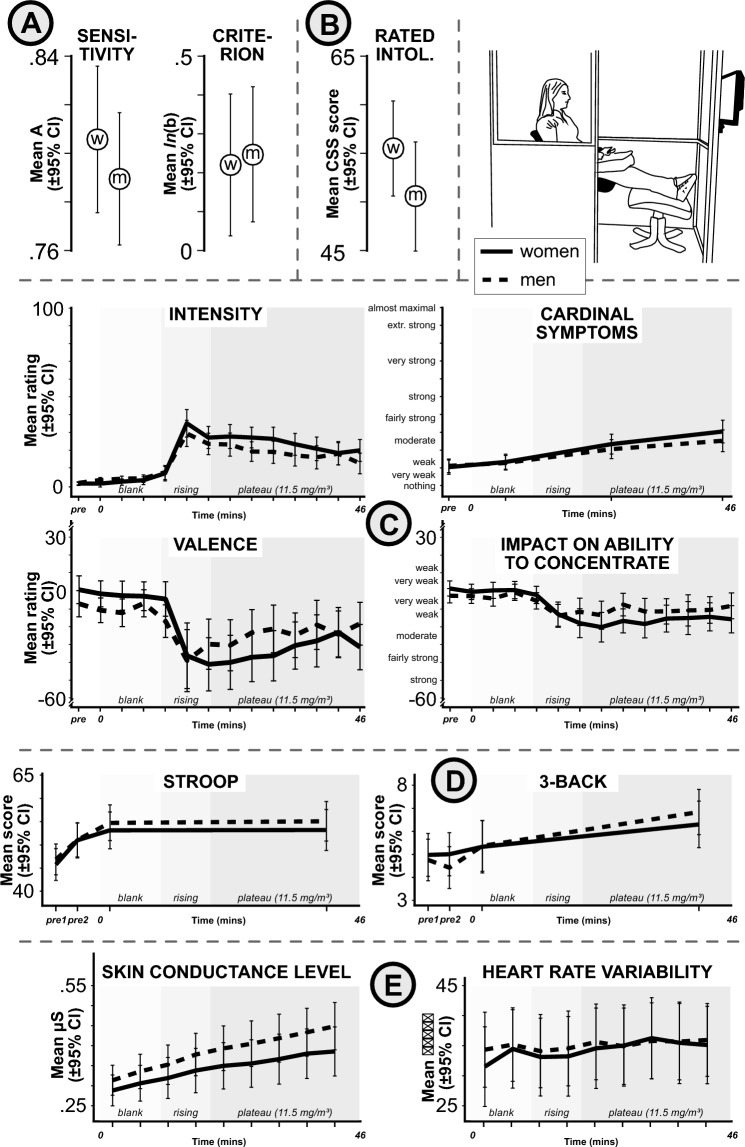
Panels depict (**A**) olfactory acuity (sensitivity; **A**) and sensory decision rule (criterion, ln(b)) assessed through a method of constant stimuli procedure in (w)omen and (m)en; (**B**) self-rated adverse reactions to odours using the Chemical Sensitivity Scale (CSS); (**C**) rated intensity, valence, impact on ability to concentrate, and cardinal symptoms following extended n-butanol exposure; (**D**) performance of Stroop and 3-back tasks; and (**E**) electrodermal activity and heart-rate variability analyzed in 5-min bins across the exposure session.

The analysis of sensitivity measure (A) was substantially in favour of H0; BF_01_ = 3.18, with a median difference of 0.16 [− 0.26, 0.58]. An additional analysis for the criterion ln(b) was also substantially in favour of H0; BF_01_ = 4.13, with similar scores for men and women (− 0.04 [− 0.46, 0.37]). Thus, results suggest that neither sensory acuity nor the sensory decision rule differed between women and men, but rather that the two sexes are similar in these regards. See Fig. 2.

### Perceptual and symptom ratings

The analysis of perceptual and symptom ratings during the extended odour exposure suggested no sex differences. The estimated sex difference for intensity ratings was -1.35 ([− 3.54, 1.13], < 1% of the rating scale) and there was substantial evidence against including the effect of Sex (BF_excl_ = 3.63). This difference was similar across all fourteen time points and there was strong evidence against including an interaction between Time and Sex. (BF_excl_ = 18.34, Table S1 in SMs). Similarly, valence ratings were similar for both men and women (0.55 [− 1.99, 3.31], < 1% of the rating scale), and there was substantial evidence against including the effect of Sex (BF_excl_ = 3.59). This difference was similar across all fourteen time points and there was anecdotal evidence against including an interaction between Time and Sex. (BF_excl_ = 1.63, Table S2 in SMs). The ability to concentrate was also similar for both men and women, with a small difference of 0.78 ([− 1.44, 3.20] < 1% of the rating scale) and there was substantial evidence against including the effect of Sex (BF_excl_ = 4.13). Again, this difference was similar across all fourteen time points and there was substantial evidence against including an interaction between Time and Sex. (BF_excl_ = 4.06, Table S3 in SMs).

The ratings of cardinal symptoms were also similar for both sexes (− 0.74 [3.45, − 1.68], < 1% of the rating scale), with substantial evidence against including the effect of Sex (BF_excl_ = 3.92). This difference was similar across all four time points and there was substantial evidence against including an interaction between Time and Sex. (BF_excl_ = 7.87, see Table S4 in SMs for all estimates).

### Cognitive tasks

The analysis of scores on cognitive tasks during the extended odour exposure suggested no sex differences. The estimated sex difference on the Stroop task was 0.38 [− 1.55, 2.23] and there was anecdotal evidence against including the effect of Sex (BF_excl_ = 2.89). This difference was similar across all four repetitions of the task and there was substantial evidence against including an interaction between Time and Sex. (BF_excl_ = 9.92, Table S5 in SMs). The scores on the 3-back tasks were also similar for both men and women (− 0.01 [− 0.47, 0.46]) and there was substantial evidence against including the effect of Sex (BF_excl_ = 6.53). This difference was similar across all four repetitions of the task and there was strong evidence against including an interaction between Time and Sex (BF_excl_ = 17.74, Table S6 in SMs).

### Autonomic nervous system recordings

The analysis of the autonomic nervous system responses during the extended odour exposure suggested no sex differences. The estimated sex difference in skin conductance was < 0.01 ([− 0.01, 0.04]) and there was anecdotal evidence against including the effect of Sex (BF_excl_ = 2.04). This difference was similar across all nine measurements and there was substantial evidence against including an interaction between Time and Sex. (BF_excl_ = 3.75, Table S7 in SMs). The heart rate variability was similar for men and women (− 0.09 [− 2.34, 1.98]) and there was anecdotal evidence against including the effect of Sex (BF_excl_ = 2.75). This difference was similar across all four repetitions of the task and there was very strong evidence against including an interaction between Time and Sex(BF_excl_ = 33.74, Table S8 in SMs).

### Self-rated chemical intolerance

We found anecdotal evidence in favour of no differences between men and women in self rated chemical intolerance (BF01 = 2.03). The ratings were similar, with a difference of − 0.26 [− 0.69, 0.16] on the total score that could range from 0 to 105.

## Discussion

The aim of this study was to investigate potential differences and similarities in reactions to odours as well as basic olfactory functions for men and women in a wide range of tests. We therefore collected measures of basic olfactory acuity (sensitivity) and sensory decision rule (criterion), perceptual ratings (intensity, valence, impact on ability to concentrate) and symptom reactions to extended olfactory exposure, performance on cognitive Stroop and 3-back, measures of autonomic nervous system activity (skin conductance level and heart-rate variability) associated with such exposure, and self-rated chemical intolerance. The sensory tests and exposure procedures thus cover outcomes not often studied when assessing possible sex/gender related differences in olfaction.

The method of constant stimulus procedure provided both a measure of olfactory acuity (sensitivity) and a decision rule when faced with sensory uncertainty (ln(b)). Analyses revealed that the results favored the null hypothesis in terms of sensitivity. Women and men were thus more similar than different in this regard, which is in line with previous studies reporting no sex-related differences in basic olfactory functioning^11,16,19–24^.

Including a measure of the decision rule adds a layer to the sensory testing by revealing how women and men deal with low-level olfactory uncertainty. A lower criterion would imply a greater propensity of reporting a stimulus as being present when uncertain, i.e., by scoring more hits but also making more false alarms. If related to everyday situations, a lower criterion could be associated with greater attention to the olfactory surroundings, but arguably also a higher risk of phantosmic experiences (i.e., perceiving a smell when no stimulus is present; see e.g., Sjölund et al^43^. However, results were in favour of a sex/gender similarity rather than a difference also in this regard.

Whole-body stimulation has been used in occupational and regulatory studies to assess reactions to ambient olfactory exposure. The method is well suited to study responses over time, and outcomes are related to how individuals rate their reactions to odours in everyday settings^44^. The current results reveal that women and men are more similar than different in how they rate and react to long-term olfactory exposure. Ratings of intensity, valence, and impact on ability to concentrate were comparable, and the sexes did not differ in terms of adverse reactions (i.e., symptoms). The results thus corroborate previous reports that women and men do not differ in their time-dependent reactions to odours^45^.

Unpleasant odours are generally regarded as distracting^46^, and having a negative bias towards an odour has been shown to worsen cognitive performance when exposed^47^. Similarly, more distressed individuals show a tendency of being more impacted by concurrent odour exposure^48^. Lower task performance during exposure could thus be an important indication of cognitive effects of odours. However, women and men performed comparably in the Stroop and 3-back tasks both before and during the exposure, which suggests that possible distracting effects of odours are similar regardless of sex.

Electrodermal activity is a measure of fluctuations in the sympathetic nervous system, and SCL is often used as a measure of tonic autonomic arousal. Heart rate variability is impacted by both the sympathetic and parasympathetic branch, and RMSSD is assumed to reflect vagal tone^49^. RMSSD has also been found to become lowered during different states of stress and distress^50^. Differences in SCL and RMSSD could thus indicate that women and men have different basic regulatory reactions when exposed. However, results revealed that the sexes were more similar than dissimilar also in this regard.

Previous reports have indicated that women report chemical intolerance to a greater degree than men do^32^, yet there are large population-based data-sets where no such effect is found^51^. Here, women and men reported similar self-rated adverse reactions to odours in everyday life. This could possibly be explained as a selection effect. There may be more women with chemical intolerance, while the majority of women and men nevertheless are similar in this regard.

Previous studies with similar exposure designs have revealed that other factors such as self-reported chemical intolerance are strong predictors for reactions. Further, individuals differed greatly in their reactions and performance, which means that the data did not lack variance to explain. Yet the two sexes reacted remarkably similar in all domains of olfaction assessed in this study. The results thus expand the literature on olfactory performance in women and men, which have predominantly utilized basic psychophysical measures of detection, discrimination and identification. If the current sample and procedure reflects everyday odour exposure situations, results favor the interpretation that women and men are more similar than different in terms of reaction and performance.

Studies on cultural or gender-based differences in how men and women relate to odours might shine a light on which factors that could be possible causes for the differences that have been observed in other studies. For instance, stereotype threat theory^29,52,53^ posits that negative stereotypes about an individual’s gender, ethnic or cultural group affect their performance on tests of abilities related to that stereotype. This theory has previously been indicated for explaining gender-based achievement gaps in e.g. mathematical ability^29,52^.

Another possibility is that women and men differ in terms of exposure and training opportunities. If olfaction is regarded as a feminine sense, men may have a disadvantage as they haven’t sought odours out to the same degree, and may thus be less attentive to odours in their everyday lives^15^. Training has been shown to have an effect on olfactory function^54–56^ even in children^57^.

The traditional view of men being less sensitive to smell than women might potentially have implications on a societal level. Preconceptions about gendered needs and behaviour could contribute to how likely a person is to seek help for a perceived medical problem^58^. A gendered expectation that men should not be sensitive to smell could make men less likely than women to bring forth issues with odourous substances in the workplace and thus suffer needlessly. This might also to some degree be a contributor to the sex disparity between woman and men reporting suffering from chemical intolerance, which is twice as common in women^12,13^ with men potentially being underdiagnosed and refraining from seeking help.

The current study does not corroborate the embryo protection theory^14,15^, as we found that women and men reacted and performed in a comparable fashion across the tests utilized in this study. However, the study is neither designed to address possible gender-related differences due to experiences, stereotype threat and similar context-dependent factors. An important issue for further studies would thus be to alter gender-related expectancies for women and men, to assess the ease of which perceptual properties of odours can be manipulated. Such studies would thus possibly provide more in-depth explanations of the source of (possible) gender differences.

## Supplementary Information

Supplementary Information.

## Data Availability

The data underlying this article will be shared on reasonable request to the corresponding author.
