# Supplementary material for: Comparable responses to a wide range of olfactory stimulation in women and men
Source: Sci Rep. 2023 Jun 3;13:9059. doi: 10.1038/s41598-023-35936-5 (PMC10239473; doi:10.1038/s41598-023-35936-5)
Supplement: Supplementary file 1 — Supplementary Information. [file 41598_2023_35936_MOESM1_ESM.docx]

**Comparable responses to a wide range of olfactory stimulation in women and men**

**Supplementary materials**

13 April, 2023

We present full results from the repeated measures ANOVA models with the effect of Time, Sex and the interaction between time and Sex.

1. Perceptual and symptom ratings

Tables S1 – S3 provide additional information on results reported in main document and show estimated differences in ratings of intensity, valence and ability to concentrate. The estimated differences are: a) ratings on each of the fourteen time points relative to mean rating (intercept), b) the overall sex difference estimate, and c) sex difference for each of the time points separately. Similarly, Table S4 contains additional information about cardinal symptoms (over four timepoint)

1. Cognitive tasks

Tables S5 – S6 provide additional information on results reported in main document and show estimated differences performance on the Stroop task, and 3-back task. The estimated differences are: a) ratings on each of the four time points relative to mean rating (intercept), b) the overall sex difference estimate, and c) sex difference for each of the time points separately.

1. Autonomic nervous system recordings

Tables S7 – S8 provide additional information on results reported in main document and show estimated differences in skin conductance levels and heart rate variability. The estimated differences are: a) ratings on each of the nine time points relative to mean rating (intercept), b) the overall sex difference estimate, and c) sex difference for each of the time points separately.

**Table S1.** Estimated intensity ratings (intercept) and time and sex related effects from the Bayesian repeated measures ANOVA including the main effects of Time and Sex, as well as their interaction. 0 – 13 – measurement time point, M – men; W – women.

| **Single Model Posterior Summary: INTENSITY** | | | | | | | | | | | |
| --- | --- | --- | --- | --- | --- | --- | --- | --- | --- | --- | --- |
|  | | | | | | | | **95% Credible Interval** | | | |
| **Variable** | | **Level** | | **Mean** | | **SD** | | **Lower** | | **Upper** | |
| Intercept |  |  |  | 15.65 |  | 1.22 |  | 13.15 |  | 17.89 |  |
| Time |  | 0 |  | -13.91 |  | 1.43 |  | -16.61 |  | -11.20 |  |
|  |  | 1 |  | -13.19 |  | 1.45 |  | -15.94 |  | -10.32 |  |
|  |  | 2 |  | -12.26 |  | 1.48 |  | -15.26 |  | -9.32 |  |
|  |  | 3 |  | -11.88 |  | 1.48 |  | -14.78 |  | -9.19 |  |
|  |  | 4 |  | -8.20 |  | 1.44 |  | -11.05 |  | -5.46 |  |
|  |  | 5 |  | 16.25 |  | 1.48 |  | 13.47 |  | 19.19 |  |
|  |  | 6 |  | 9.40 |  | 1.53 |  | 6.43 |  | 12.51 |  |
|  |  | 7 |  | 9.57 |  | 1.43 |  | 6.61 |  | 12.24 |  |
|  |  | 8 |  | 7.28 |  | 1.44 |  | 4.60 |  | 10.10 |  |
|  |  | 9 |  | 6.88 |  | 1.44 |  | 4.02 |  | 9.65 |  |
|  |  | 10 |  | 4.42 |  | 1.48 |  | 1.51 |  | 7.27 |  |
|  |  | 11 |  | 2.65 |  | 1.47 |  | -0.31 |  | 5.45 |  |
|  |  | 12 |  | 2.46 |  | 1.42 |  | -0.22 |  | 5.23 |  |
|  |  | 13 |  | 0.53 |  | 1.48 |  | -2.52 |  | 3.28 |  |
| Sex |  | M |  | -1.35 |  | 1.19 |  | -3.54 |  | 1.13 |  |
|  |  | W |  | 1.35 |  | 1.19 |  | -1.13 |  | 3.54 |  |
| Time ✻  Sex |  | 0 (M) |  | 0.99 |  | 1.21 |  | -1.26 |  | 3.40 |  |
|  |  | 0 (W) |  | -0.99 |  | 1.21 |  | -3.40 |  | 1.26 |  |
|  |  | 1 (M) |  | 1.72 |  | 1.24 |  | -0.69 |  | 4.14 |  |
|  |  | 1 (W) |  | -1.72 |  | 1.24 |  | -4.14 |  | 0.69 |  |
|  |  | 2 (M) |  | 1.57 |  | 1.19 |  | -0.73 |  | 3.92 |  |
|  |  | 2 (W) |  | -1.57 |  | 1.19 |  | -3.92 |  | 0.73 |  |
|  |  | 3 (M) |  | 1.32 |  | 1.20 |  | -1.04 |  | 4.02 |  |
|  |  | 3 (W) |  | -1.32 |  | 1.20 |  | -4.02 |  | 1.04 |  |
|  |  | 4 (M) |  | 1.17 |  | 1.21 |  | -1.12 |  | 3.57 |  |
|  |  | 4 (W) |  | -1.17 |  | 1.21 |  | -3.57 |  | 1.12 |  |
|  |  | 5 (M) |  | -0.86 |  | 1.23 |  | -3.36 |  | 1.44 |  |
|  |  | 5 (W) |  | 0.86 |  | 1.23 |  | -1.44 |  | 3.36 |  |
|  |  | 6 (M) |  | -0.14 |  | 1.19 |  | -2.56 |  | 2.09 |  |
|  |  | 6 (W) |  | 0.14 |  | 1.19 |  | -2.09 |  | 2.56 |  |
|  |  | 7 (M) |  | -0.53 |  | 1.15 |  | -2.94 |  | 1.64 |  |
|  |  | 7 (W) |  | 0.53 |  | 1.15 |  | -1.64 |  | 2.94 |  |
|  |  | 8 (M) |  | -1.60 |  | 1.26 |  | -4.32 |  | 0.80 |  |
|  |  | 8 (W) |  | 1.60 |  | 1.26 |  | -0.80 |  | 4.32 |  |
|  |  | 9 (M) |  | -1.41 |  | 1.26 |  | -3.84 |  | 0.99 |  |
|  |  | 9 (W) |  | 1.41 |  | 1.26 |  | -0.99 |  | 3.84 |  |
|  |  | 10 (M) |  | -1.04 |  | 1.19 |  | -3.39 |  | 1.24 |  |
|  |  | 10 (W) |  | 1.04 |  | 1.19 |  | -1.24 |  | 3.39 |  |
|  |  | 11 (M) |  | -0.58 |  | 1.23 |  | -2.97 |  | 1.81 |  |
|  |  | 11 (W) |  | 0.58 |  | 1.23 |  | -1.81 |  | 2.97 |  |
|  |  | 12 (M) |  | 0.85 |  | 1.20 |  | -1.52 |  | 3.17 |  |
|  |  | 12 (W) |  | -0.85 |  | 1.20 |  | -3.17 |  | 1.52 |  |
|  |  | 13 (M) |  | -1.46 |  | 1.19 |  | -3.89 |  | 0.78 |  |
|  |  | 13 (W) |  | 1.46 |  | 1.19 |  | -0.78 |  | 3.89 |  |
|  | | | | | | | | | | | |

**Table S2.** Estimated valence ratings (intercept) and time and sex related effects from the Bayesian repeated measures ANOVA including the main effects of Time and Sex, as well as their interaction. 0 – 13 – measurement time point; M – men; W – women.

| **Single Model Posterior Summary: VALENCE** | | | | | | | | | | | |
| --- | --- | --- | --- | --- | --- | --- | --- | --- | --- | --- | --- |
|  | | | | | | | | **95% Credible Interval** | | | |
| **Variable** | | **Level** | | **Mean** | | **SD** | | **Lower** | | **Upper** | |
| Intercept |  |  |  | -5.86 |  | 1.47 |  | -8.78 |  | -3.04 |  |
| Time |  | 0 |  | 8.45 |  | 1.60 |  | 5.38 |  | 11.50 |  |
|  |  | 1 |  | 7.17 |  | 1.57 |  | 4.11 |  | 10.19 |  |
|  |  | 2 |  | 6.40 |  | 1.63 |  | 3.11 |  | 9.48 |  |
|  |  | 3 |  | 7.62 |  | 1.55 |  | 4.51 |  | 10.55 |  |
|  |  | 4 |  | 5.00 |  | 1.55 |  | 2.00 |  | 7.89 |  |
|  |  | 5 |  | -7.64 |  | 1.58 |  | -10.80 |  | -4.60 |  |
|  |  | 6 |  | -6.51 |  | 1.60 |  | -9.80 |  | -3.18 |  |
|  |  | 7 |  | -6.44 |  | 1.55 |  | -9.60 |  | -3.50 |  |
|  |  | 8 |  | -4.09 |  | 1.66 |  | -7.45 |  | -0.79 |  |
|  |  | 9 |  | -3.36 |  | 1.61 |  | -6.51 |  | -0.16 |  |
|  |  | 10 |  | -2.92 |  | 1.52 |  | -5.90 |  | -0.14 |  |
|  |  | 11 |  | -0.95 |  | 1.62 |  | -4.22 |  | 2.05 |  |
|  |  | 12 |  | -1.14 |  | 1.53 |  | -4.10 |  | 1.84 |  |
|  |  | 13 |  | -1.58 |  | 1.52 |  | -4.44 |  | 1.27 |  |
| Sex |  | M |  | 0.55 |  | 1.37 |  | -1.99 |  | 3.31 |  |
|  |  | W |  | -0.55 |  | 1.37 |  | -3.31 |  | 1.99 |  |
| Time ✻  Sex |  | 0 (M) |  | -1.74 |  | 1.43 |  | -4.60 |  | 1.04 |  |
|  |  | 0 (W) |  | 1.74 |  | 1.43 |  | -1.04 |  | 4.60 |  |
|  |  | 1 (M) |  | -1.80 |  | 1.39 |  | -4.67 |  | 0.88 |  |
|  |  | 1 (W) |  | 1.80 |  | 1.39 |  | -0.88 |  | 4.67 |  |
|  |  | 2 (M) |  | -2.01 |  | 1.32 |  | -4.57 |  | 0.61 |  |
|  |  | 2 (W) |  | 2.01 |  | 1.32 |  | -0.61 |  | 4.57 |  |
|  |  | 3 (M) |  | -1.02 |  | 1.37 |  | -3.83 |  | 1.64 |  |
|  |  | 3 (W) |  | 1.02 |  | 1.37 |  | -1.64 |  | 3.83 |  |
|  |  | 4 (M) |  | -2.66 |  | 1.44 |  | -5.59 |  | 0.03 |  |
|  |  | 4 (W) |  | 2.66 |  | 1.44 |  | -0.03 |  | 5.59 |  |
|  |  | 5 (M) |  | -0.86 |  | 1.37 |  | -3.60 |  | 1.82 |  |
|  |  | 5 (W) |  | 0.86 |  | 1.37 |  | -1.82 |  | 3.60 |  |
|  |  | 6 (M) |  | 1.47 |  | 1.35 |  | -0.99 |  | 4.20 |  |
|  |  | 6 (W) |  | -1.47 |  | 1.35 |  | -4.20 |  | 0.99 |  |
|  |  | 7 (M) |  | 1.26 |  | 1.38 |  | -1.41 |  | 4.01 |  |
|  |  | 7 (W) |  | -1.26 |  | 1.38 |  | -4.01 |  | 1.41 |  |
|  |  | 8 (M) |  | 1.98 |  | 1.36 |  | -0.44 |  | 4.67 |  |
|  |  | 8 (W) |  | -1.98 |  | 1.36 |  | -4.67 |  | 0.44 |  |
|  |  | 9 (M) |  | 2.27 |  | 1.39 |  | -0.30 |  | 5.12 |  |
|  |  | 9 (W) |  | -2.27 |  | 1.39 |  | -5.12 |  | 0.30 |  |
|  |  | 10 (M) |  | 0.65 |  | 1.34 |  | -2.02 |  | 3.30 |  |
|  |  | 10 (W) |  | -0.65 |  | 1.34 |  | -3.30 |  | 2.02 |  |
|  |  | 11 (M) |  | 1.14 |  | 1.38 |  | -1.37 |  | 3.77 |  |
|  |  | 11 (W) |  | -1.14 |  | 1.38 |  | -3.77 |  | 1.37 |  |
|  |  | 12 (M) |  | -0.52 |  | 1.33 |  | -3.14 |  | 2.19 |  |
|  |  | 12 (W) |  | 0.52 |  | 1.33 |  | -2.19 |  | 3.14 |  |
|  |  | 13 (M) |  | 1.82 |  | 1.40 |  | -0.96 |  | 4.70 |  |
|  |  | 13 (W) |  | -1.82 |  | 1.40 |  | -4.70 |  | 0.96 |  |
|  | | | | | | | | | | | |

**Table S3.** Estimated ability to concentrate ratings (intercept) and time and sex related effects from the Bayesian repeated measures ANOVA including the main effects of Time and Sex, as well as their interaction. 0 – 13 – measurement time point; M – men; W – women.

| **Single Model Posterior Summary: ABILITY TO CONCENTRATE** | | | | | | | | | | | |
| --- | --- | --- | --- | --- | --- | --- | --- | --- | --- | --- | --- |
|  | | | | | | | | **95% Credible Interval** | | | |
| **Variable** | | **Level** | | **Mean** | | **SD** | | **Lower** | | **Upper** | |
| Intercept |  |  |  | -4.79 |  | 1.28 |  | -7.45 |  | -2.34 |  |
| Time |  | 0 |  | 6.08 |  | 1.22 |  | 3.67 |  | 8.41 |  |
|  |  | 1 |  | 5.38 |  | 1.23 |  | 2.95 |  | 7.72 |  |
|  |  | 2 |  | 5.21 |  | 1.20 |  | 2.83 |  | 7.55 |  |
|  |  | 3 |  | 6.57 |  | 1.25 |  | 4.17 |  | 8.95 |  |
|  |  | 4 |  | 3.88 |  | 1.19 |  | 1.49 |  | 6.30 |  |
|  |  | 5 |  | -2.81 |  | 1.22 |  | -5.21 |  | -0.42 |  |
|  |  | 6 |  | -3.80 |  | 1.24 |  | -6.31 |  | -1.42 |  |
|  |  | 7 |  | -5.23 |  | 1.23 |  | -7.68 |  | -2.94 |  |
|  |  | 8 |  | -1.87 |  | 1.25 |  | -4.44 |  | 0.51 |  |
|  |  | 9 |  | -4.00 |  | 1.20 |  | -6.29 |  | -1.63 |  |
|  |  | 10 |  | -2.80 |  | 1.25 |  | -5.26 |  | -0.28 |  |
|  |  | 11 |  | -2.55 |  | 1.27 |  | -4.99 |  | -0.13 |  |
|  |  | 12 |  | -2.20 |  | 1.23 |  | -4.62 |  | 0.24 |  |
|  |  | 13 |  | -1.86 |  | 1.23 |  | -4.26 |  | 0.60 |  |
| Sex |  | M |  | 0.78 |  | 1.16 |  | -1.44 |  | 3.20 |  |
|  |  | W |  | -0.78 |  | 1.16 |  | -3.20 |  | 1.44 |  |
| Time ✻  Sex |  | 0 (M) |  | -1.67 |  | 1.06 |  | -3.93 |  | 0.29 |  |
|  |  | 0 (W) |  | 1.67 |  | 1.06 |  | -0.29 |  | 3.93 |  |
|  |  | 1 (M) |  | -1.18 |  | 1.13 |  | -3.38 |  | 1.04 |  |
|  |  | 1 (W) |  | 1.18 |  | 1.13 |  | -1.04 |  | 3.38 |  |
|  |  | 2 (M) |  | -1.77 |  | 1.08 |  | -3.84 |  | 0.24 |  |
|  |  | 2 (W) |  | 1.77 |  | 1.08 |  | -0.24 |  | 3.84 |  |
|  |  | 3 (M) |  | -0.95 |  | 1.10 |  | -3.13 |  | 1.23 |  |
|  |  | 3 (W) |  | 0.95 |  | 1.10 |  | -1.23 |  | 3.13 |  |
|  |  | 4 (M) |  | -1.48 |  | 1.09 |  | -3.59 |  | 0.55 |  |
|  |  | 4 (W) |  | 1.48 |  | 1.09 |  | -0.55 |  | 3.59 |  |
|  |  | 5 (M) |  | -0.86 |  | 1.10 |  | -2.99 |  | 1.33 |  |
|  |  | 5 (W) |  | 0.86 |  | 1.10 |  | -1.33 |  | 2.99 |  |
|  |  | 6 (M) |  | 1.12 |  | 1.08 |  | -1.05 |  | 3.15 |  |
|  |  | 6 (W) |  | -1.12 |  | 1.08 |  | -3.15 |  | 1.05 |  |
|  |  | 7 (M) |  | 1.12 |  | 1.04 |  | -1.05 |  | 3.17 |  |
|  |  | 7 (W) |  | -1.12 |  | 1.04 |  | -3.17 |  | 1.05 |  |
|  |  | 8 (M) |  | 1.76 |  | 1.10 |  | -0.29 |  | 3.97 |  |
|  |  | 8 (W) |  | -1.76 |  | 1.10 |  | -3.97 |  | 0.29 |  |
|  |  | 9 (M) |  | 1.18 |  | 1.04 |  | -0.95 |  | 3.27 |  |
|  |  | 9 (W) |  | -1.18 |  | 1.04 |  | -3.27 |  | 0.95 |  |
|  |  | 10 (M) |  | 0.49 |  | 1.06 |  | -1.61 |  | 2.54 |  |
|  |  | 10 (W) |  | -0.49 |  | 1.06 |  | -2.54 |  | 1.61 |  |
|  |  | 11 (M) |  | 0.49 |  | 1.07 |  | -1.65 |  | 2.57 |  |
|  |  | 11 (W) |  | -0.49 |  | 1.07 |  | -2.57 |  | 1.65 |  |
|  |  | 12 (M) |  | 0.39 |  | 1.08 |  | -1.78 |  | 2.56 |  |
|  |  | 12 (W) |  | -0.39 |  | 1.08 |  | -2.56 |  | 1.78 |  |
|  |  | 13 (M) |  | 1.37 |  | 1.10 |  | -0.83 |  | 3.53 |  |
|  |  | 13 (W) |  | -1.37 |  | 1.10 |  | -3.53 |  | 0.83 |  |
|  | | | | | | | | | | | |

**Table S4.** Estimated cardinal symptom ratings (intercept) and time and sex related effects from the Bayesian repeated measures ANOVA including the main effects of Time and Sex, as well as their interaction. 0 – 3 - measurement time point; M – men; W – women.

| **Single Model Posterior Summary : CARDINAL SYMPTOM** | | | | | | | | | | | |
| --- | --- | --- | --- | --- | --- | --- | --- | --- | --- | --- | --- |
|  | | | | | | | | **95% Credible Interval** | | | |
| **Variable** | | **Level** | | **Mean** | | **SD** | | **Lower** | | **Upper** | |
| Intercept |  |  |  | 18.51 |  | 1.53 |  | 15.50 |  | 21.67 |  |
| Time |  | 0 |  | -7.30 |  | 1.06 |  | -9.40 |  | -5.23 |  |
|  |  | 1 |  | -5.05 |  | 1.08 |  | -7.10 |  | -2.88 |  |
|  |  | 2 |  | 3.35 |  | 1.03 |  | 1.40 |  | 5.54 |  |
|  |  | 3 |  | 8.99 |  | 1.10 |  | 6.88 |  | 11.11 |  |
| Sex |  | M |  | -0.74 |  | 1.32 |  | -3.45 |  | 1.68 |  |
|  |  | W |  | 0.74 |  | 1.32 |  | -1.68 |  | 3.45 |  |
| Time ✻  Sex |  | 0 (M) |  | 1.10 |  | 0.97 |  | -0.77 |  | 3.14 |  |
|  |  | 0 (W) |  | -1.10 |  | 0.97 |  | -3.14 |  | 0.77 |  |
|  |  | 1 (M) |  | 0.47 |  | 0.94 |  | -1.43 |  | 2.28 |  |
|  |  | 1 (W) |  | -0.47 |  | 0.94 |  | -2.28 |  | 1.43 |  |
|  |  | 2 (M) |  | -0.29 |  | 0.90 |  | -2.04 |  | 1.45 |  |
|  |  | 2 (W) |  | 0.29 |  | 0.90 |  | -1.45 |  | 2.04 |  |
|  |  | 3 (M) |  | -1.27 |  | 0.94 |  | -3.18 |  | 0.48 |  |
|  |  | 3 (W) |  | 1.27 |  | 0.94 |  | -0.48 |  | 3.18 |  |
|  | | | | | | | | | | | |

**Table S5.** Estimated performance on the Stroop task (intercept) and time and sex related effects from the Bayesian repeated measures ANOVA including the main effects of Time and Sex, as well as their interaction. 0 – 3 - measurement time point; M – men; W – women.

| **Single Model Posterior Summary: STROOP TASK** | | | | | | | | | | | |
| --- | --- | --- | --- | --- | --- | --- | --- | --- | --- | --- | --- |
|  | | | | | | | | **95% Credible Interval** | | | |
| **Variable** | | **Level** | | **Mean** | | **SD** | | **Lower** | | **Upper** | |
| Intercept |  |  |  | 51.37 |  | 1.22 |  | 49.03 |  | 53.66 |  |
| Time |  | 0 |  | -4.90 |  | 0.51 |  | -5.90 |  | -3.84 |  |
|  |  | 1 |  | -0.30 |  | 0.48 |  | -1.28 |  | 0.62 |  |
|  |  | 2 |  | 2.51 |  | 0.50 |  | 1.45 |  | 3.44 |  |
|  |  | 3 |  | 2.69 |  | 0.52 |  | 1.66 |  | 3.65 |  |
| Sex |  | M |  | 0.38 |  | 0.98 |  | -1.55 |  | 2.23 |  |
|  |  | W |  | -0.38 |  | 0.98 |  | -2.23 |  | 1.55 |  |
| Time ✻  Sex |  | 0 (M) |  | -8.45×10^-3^ |  | 0.44 |  | -0.95 |  | 0.83 |  |
|  |  | 0 (W) |  | 8.45×10^-3^ |  | 0.44 |  | -0.83 |  | 0.95 |  |
|  |  | 1 (M) |  | -0.43 |  | 0.44 |  | -1.31 |  | 0.41 |  |
|  |  | 1 (W) |  | 0.43 |  | 0.44 |  | -0.41 |  | 1.31 |  |
|  |  | 2 (M) |  | 0.17 |  | 0.46 |  | -0.72 |  | 1.10 |  |
|  |  | 2 (W) |  | -0.17 |  | 0.46 |  | -1.10 |  | 0.72 |  |
|  |  | 3 (M) |  | 0.27 |  | 0.45 |  | -0.59 |  | 1.15 |  |
|  |  | 3 (W) |  | -0.27 |  | 0.45 |  | -1.15 |  | 0.59 |  |
|  | | | | | | | | | | | |

**Table S6.** Estimated performance on the 3-back task (intercept) and time and sex related effects from the Bayesian repeated measures ANOVA including the main effects of Time and Sex, as well as their interaction. 0 – 3 - measurement time point; M – men; W – women.

| **Single Model Posterior Summary: 3-BACK TASK** | | | | | | | | | | | |
| --- | --- | --- | --- | --- | --- | --- | --- | --- | --- | --- | --- |
|  | | | | | | | | **95% Credible Interval** | | | |
| **Variable** | | **Level** | | **Mean** | | **SD** | | **Lower** | | **Upper** | |
| Intercept |  |  |  | 5.37 |  | 0.25 |  | 4.91 |  | 5.89 |  |
| Time |  | 0 |  | -0.47 |  | 0.24 |  | -0.95 |  | -7.60×10^-3^ |  |
|  |  | 1 |  | -0.60 |  | 0.24 |  | -1.09 |  | -0.10 |  |
|  |  | 2 |  | -7.38×10^-3^ |  | 0.23 |  | -0.47 |  | 0.45 |  |
|  |  | 3 |  | 1.08 |  | 0.25 |  | 0.60 |  | 1.59 |  |
| Sex |  | M |  | -0.01 |  | 0.24 |  | -0.47 |  | 0.46 |  |
|  |  | W |  | 0.01 |  | 0.24 |  | -0.46 |  | 0.47 |  |
| Time ✻  Sex |  | 0 (M) |  | -0.07 |  | 0.22 |  | -0.49 |  | 0.37 |  |
|  |  | 0 (W) |  | 0.07 |  | 0.22 |  | -0.37 |  | 0.49 |  |
|  |  | 1 (M) |  | -0.20 |  | 0.23 |  | -0.67 |  | 0.25 |  |
|  |  | 1 (W) |  | 0.20 |  | 0.23 |  | -0.25 |  | 0.67 |  |
|  |  | 2 (M) |  | 0.04 |  | 0.21 |  | -0.40 |  | 0.44 |  |
|  |  | 2 (W) |  | -0.04 |  | 0.21 |  | -0.44 |  | 0.40 |  |
|  |  | 3 (M) |  | 0.23 |  | 0.22 |  | -0.20 |  | 0.69 |  |
|  |  | 3 (W) |  | -0.23 |  | 0.22 |  | -0.69 |  | 0.20 |  |
|  | | | | | | | | | | | |

| **Table S7.** Estimated skin conductance levels (intercept) and time and sex related effects from the Bayesian repeated measures ANOVA including the main effects of Time and Sex, as well as their interaction. 1 – 9 - measurement time point; M – men; W – women.  **Single Model Posterior Summary: SKIN CONDUCTANCE** | | | | | | | | | | | |
| --- | --- | --- | --- | --- | --- | --- | --- | --- | --- | --- | --- |
|  | | | | | | | | **95% Credible Interval** | | | |
| **Variable** | | **Level** | | **Mean** | | **SD** | | **Lower** | | **Upper** | |
| Intercept |  |  |  | 0.37 |  | 0.02 |  | 0.33 |  | 0.40 |  |
| Time |  | 1 |  | -0.06 |  | 4.85×10^-3^ |  | -0.07 |  | -0.05 |  |
|  |  | 2 |  | -0.04 |  | 4.78×10^-3^ |  | -0.05 |  | -0.03 |  |
|  |  | 3 |  | -0.03 |  | 4.62×10^-3^ |  | -0.04 |  | -0.02 |  |
|  |  | 4 |  | -6.73×10^-3^ |  | 5.54×10^-3^ |  | -0.02 |  | 1.50×10^-3^ |  |
|  |  | 5 |  | 6.58×10^-3^ |  | 4.63×10^-3^ |  | -2.28×10^-3^ |  | 0.02 |  |
|  |  | 6 |  | 0.02 |  | 5.55×10^-3^ |  | 6.29×10^-3^ |  | 0.02 |  |
|  |  | 7 |  | 0.03 |  | 7.37×10^-3^ |  | 0.02 |  | 0.04 |  |
|  |  | 8 |  | 0.04 |  | 4.96×10^-3^ |  | 0.03 |  | 0.05 |  |
|  |  | 9 |  | 0.05 |  | 4.62×10^-3^ |  | 0.04 |  | 0.06 |  |
| Sex |  | M |  | 9.83×10^-3^ |  | 0.01 |  | -0.01 |  | 0.04 |  |
|  |  | W |  | -9.83×10^-3^ |  | 0.01 |  | -0.04 |  | 0.01 |  |
| Time ✻  Sex |  | 1 (M) |  | -6.54×10^-3^ |  | 4.18×10^-3^ |  | -0.01 |  | 1.13×10^-3^ |  |
|  |  | 1 (W) |  | 6.54×10^-3^ |  | 4.18×10^-3^ |  | -1.13×10^-3^ |  | 0.01 |  |
|  |  | 2 (M) |  | -5.03×10^-3^ |  | 4.16×10^-3^ |  | -0.01 |  | 2.19×10^-3^ |  |
|  |  | 2 (W) |  | 5.03×10^-3^ |  | 4.16×10^-3^ |  | -2.19×10^-3^ |  | 0.01 |  |
|  |  | 3 (M) |  | -3.68×10^-3^ |  | 4.26×10^-3^ |  | -0.01 |  | 3.72×10^-3^ |  |
|  |  | 3 (W) |  | 3.68×10^-3^ |  | 4.26×10^-3^ |  | -3.72×10^-3^ |  | 0.01 |  |
|  |  | 4 (M) |  | -8.61×10^-4^ |  | 4.22×10^-3^ |  | -8.24×10^-3^ |  | 7.14×10^-3^ |  |
|  |  | 4 (W) |  | 8.61×10^-4^ |  | 4.22×10^-3^ |  | -7.14×10^-3^ |  | 8.24×10^-3^ |  |
|  |  | 5 (M) |  | -1.07×10^-4^ |  | 5.45×10^-3^ |  | -7.27×10^-3^ |  | 7.67×10^-3^ |  |
|  |  | 5 (W) |  | 1.07×10^-4^ |  | 5.45×10^-3^ |  | -7.67×10^-3^ |  | 7.27×10^-3^ |  |
|  |  | 6 (M) |  | 1.99×10^-3^ |  | 4.24×10^-3^ |  | -6.01×10^-3^ |  | 9.82×10^-3^ |  |
|  |  | 6 (W) |  | -1.99×10^-3^ |  | 4.24×10^-3^ |  | -9.82×10^-3^ |  | 6.01×10^-3^ |  |
|  |  | 7 (M) |  | 3.35×10^-3^ |  | 4.07×10^-3^ |  | -3.69×10^-3^ |  | 0.01 |  |
|  |  | 7 (W) |  | -3.35×10^-3^ |  | 4.07×10^-3^ |  | -0.01 |  | 3.69×10^-3^ |  |
|  |  | 8 (M) |  | 3.94×10^-3^ |  | 4.81×10^-3^ |  | -3.58×10^-3^ |  | 0.01 |  |
|  |  | 8 (W) |  | -3.94×10^-3^ |  | 4.81×10^-3^ |  | -0.01 |  | 3.58×10^-3^ |  |
|  |  | 9 (M) |  | 6.93×10^-3^ |  | 6.76×10^-3^ |  | -4.42×10^-4^ |  | 0.01 |  |
|  |  | 9 (W) |  | -6.93×10^-3^ |  | 6.76×10^-3^ |  | -0.01 |  | 4.42×10^-4^ |  |
|  | | | | | | | | | | | |

| **Table S8.** Estimated heart rate variability (intercept) and time and sex related effects from the Bayesian repeated measures ANOVA including the main effects of Time and Sex, as well as their interaction. 1 – 9 - measurement time point; M – men; W – women.  **Single Model Posterior Summary: HEART RATE VARIABILITY** | | | | | | | | | | | |
| --- | --- | --- | --- | --- | --- | --- | --- | --- | --- | --- | --- |
|  | | | | | | | | **95% Credible Interval** | | | |
| **Variable** | | **Level** | | **Mean** | | **SD** | | **Lower** | | **Upper** | |
| Intercept |  |  |  | 76.93 |  | 1.52 |  | 74.05 |  | 80.14 |  |
| Time |  | 1 |  | 4.31 |  | 0.54 |  | 3.25 |  | 5.38 |  |
|  |  | 2 |  | 0.64 |  | 0.51 |  | -0.35 |  | 1.60 |  |
|  |  | 3 |  | -0.33 |  | 0.50 |  | -1.34 |  | 0.63 |  |
|  |  | 4 |  | -0.22 |  | 0.51 |  | -1.22 |  | 0.71 |  |
|  |  | 5 |  | -0.36 |  | 0.51 |  | -1.33 |  | 0.61 |  |
|  |  | 6 |  | -0.99 |  | 0.50 |  | -1.99 |  | -0.06 |  |
|  |  | 7 |  | -0.84 |  | 0.49 |  | -1.83 |  | 0.09 |  |
|  |  | 8 |  | -0.71 |  | 0.50 |  | -1.71 |  | 0.34 |  |
|  |  | 9 |  | -1.48 |  | 0.49 |  | -2.53 |  | -0.55 |  |
| Sex |  | M |  | -0.09 |  | 1.10 |  | -2.34 |  | 1.98 |  |
|  |  | W |  | 0.09 |  | 1.10 |  | -1.98 |  | 2.34 |  |
| Time ✻  Sex |  | 1 (M) |  | -0.24 |  | 0.43 |  | -1.13 |  | 0.55 |  |
|  |  | 1 (W) |  | 0.24 |  | 0.43 |  | -0.55 |  | 1.13 |  |
|  |  | 2 (M) |  | 0.12 |  | 0.44 |  | -0.77 |  | 0.97 |  |
|  |  | 2 (W) |  | -0.12 |  | 0.44 |  | -0.97 |  | 0.77 |  |
|  |  | 3 (M) |  | -0.08 |  | 0.42 |  | -0.93 |  | 0.70 |  |
|  |  | 3 (W) |  | 0.08 |  | 0.42 |  | -0.70 |  | 0.93 |  |
|  |  | 4 (M) |  | -0.11 |  | 0.42 |  | -0.96 |  | 0.70 |  |
|  |  | 4 (W) |  | 0.11 |  | 0.42 |  | -0.70 |  | 0.96 |  |
|  |  | 5 (M) |  | -0.23 |  | 0.42 |  | -1.06 |  | 0.61 |  |
|  |  | 5 (W) |  | 0.23 |  | 0.42 |  | -0.61 |  | 1.06 |  |
|  |  | 6 (M) |  | -0.35 |  | 0.43 |  | -1.25 |  | 0.47 |  |
|  |  | 6 (W) |  | 0.35 |  | 0.43 |  | -0.47 |  | 1.25 |  |
|  |  | 7 (M) |  | -0.25 |  | 0.45 |  | -1.13 |  | 0.63 |  |
|  |  | 7 (W) |  | 0.25 |  | 0.45 |  | -0.63 |  | 1.13 |  |
|  |  | 8 (M) |  | 0.59 |  | 0.44 |  | -0.27 |  | 1.49 |  |
|  |  | 8 (W) |  | -0.59 |  | 0.44 |  | -1.49 |  | 0.27 |  |
|  |  | 9 (M) |  | 0.56 |  | 0.44 |  | -0.26 |  | 1.45 |  |
|  |  | 9 (W) |  | -0.56 |  | 0.44 |  | -1.45 |  | 0.26 |  |
|  | | | | | | | | | | | |
